# Therapeutic effects of moxibustion simultaneously targeting *Nrf2* and *NF-κB* in diabetic peripheral neuropathy

**DOI:** 10.1007/s12010-019-03052-8

**Published:** 2019-06-17

**Authors:** Jia Li, Xia Hu, Fengxia Liang, Jianmin Liu, Huanjiao Zhou, Jiaoping Liu, Hua Wang, Hongtu Tang

**Affiliations:** 1 Hubei University of Chinese Medicine/Hubei Provincial Collaborative Innovation Center of Preventive Treatment by Acupuncture and Moxibustion, Wuhan, 430061 China; 2 College of Basic Medicine, Hubei University of Chinese Medicine, Wuhan, 430061 China; 3 Department of Oncology, Integrated Chinese and Western Medicines, The Central Hospital of Wuhan, Tongji Medical College, Huazhong University of Science & Technology (HUST), Wuhan, 430010 China

**Keywords:** Diabetic peripheral neuropathy, Moxibustion, Neuroinflammation, Cytokine

## Abstract

Moxibustion is the main alternative medicine treatment that has been beneficial to diabetic peripheral neuropathy (DPN), a common complication secondary to diabetic microvascular injury. However, the underlying protective mechanism of moxibustion against neuroinflammation remains unclear. We hypothesized that moxibustion treats DPN by regulating the balance of nuclear factor-2 erythroid-related factor-2 (*Nrf2*)-nuclear factor-kappa light chain enhancer of B cells (*NF-кB*). In vivo, diabetes was induced in rats by injecting streptozotocin (STZ; 60 mg/kg; i.p.). Moxibustion was then applied to “Zusanli” (ST 36), “Guanyuan” (BL 26), and “Yishu” (EX-B 3) acupuncture points. Nerve conduction was detected. Serum interleukin (IL)-1β, IL-6, and IL-8 levels were determined through enzyme-linked immunosorbent assay. *NF-κB* and *Nrf2* proteins were examined through immunoblot analysis. The mRNA of *NF-κB* and *Nrf2* was evaluated through RT-PCR. We found that the conduction velocity and amplitude of the action potentials of sciatic nerve conduction were reduced in the DPN model group but were rescued by moxibustion treatment. Moxibustion also improved the effect of DPN on other parameters, including ultrastructural changes, *NF-κB* and *Nrf2* expression in the sciatic nerve, and serum IL-1β, IL-6, and IL-8 levels. Our data suggested that moxibustion may alleviate neuroinflammation by inhibiting *NF-κB* and by activating *Nrf2*. Moxibustion may also provide therapeutic effects for patients with DPN by simultaneously targeting *Nrf2* and *NF-κB*.

## Background

According to the World Health Organization (WHO), the prevalence of diabetes mellitus is predicted to increase by 170% between 1995 and 2025 [1]; furthermore, 353 million individuals will suffer from diabetes mellitus by 2030 [2]. Diabetic neuropathy is the most common complication associated with peripheral neuropathy; this condition may be caused by diseases, nerve trauma, or side effects induced by systemic illness [3]. Diabetic neuropathy also results in large disease burdens, such as incapacity to work, poor quality of life, and consumption of health care resources [4]. However, the etiology of diabetic neuropathy is poorly understood. Although diabetic neuropathy is treated through glycemic control and diligent foot care, other treatments have yet to be developed. Therapeutic methods, including those currently evaluated through clinical trials, have been used to treat diabetic peripheral neuropathy (DPN) with limited efficacy. DPN is mainly prevented by glycemic control or proper pathological management; nevertheless, these strategies are poorly implemented [5, 6]. To relieve the pain associated with DPN, researchers should investigate common pharmacological treatments used to respond to the poor effect of common analgesics, such as non-steroidal anti-inflammatory drugs (NSAIDS) and opioids or gabapentin versus pregabalin clinical use; researchers should also describe Oriental Medicine guidelines and a long list of natural compounds identified as possible therapeutic or alternative agents to replace or to combine with existing therapies [7].

Acupuncture and moxibustion have experienced widespread adoption worldwide and their popularity has seen a gradual rise, but in China, they have a long history of over 4,000 years. Moxibustion is a therapy that treats and prevents diseases using moxa floss. As an external treatment to treat or prevent diseases and promote health of the body, the combustion of the moxa floss permits transmission of thermal effect on certain points or areas of the body surface to treat disease. Acupuncture methods are widely applied to treat DPN on the basis of research on clinical curative effects [8–10] and experimental mechanisms [11]. The effectiveness of acupuncture points has also been confirmed. Thai foot massage using deep pressure and stretching can improve foot sensation in DPN patients after a 2-week treatment [12]. One RCT suggested that electroacupuncture is more effective in improving physical activity and QoL and reduces the need for oral analgesic medication than manual acupuncture [13]. Likewise, moxibustion treatment is used to clinically treat arthritis [14], knee osteoarthritis [15, 16], and diabetes [17, 18]. Previous studies have assessed acupuncture’s preventive effect on DPN by improving the morphology and function of the myelinated fiber of the sciatic nerve. Garrow et al [19] have published the only randomized, placebo-controlled study of acupuncture in the management of lower limb DPN, reporting analgesic effect in the acupuncture group compared with little change in the sham group. Abuaisha et al [20] also reported nerve conduction velocity and a variety of subjective symptoms associated with this progressive disabling disorder improvement before and after acupuncture treatment. Tong et al [21] found significant improvements in both numbness in the upper extremities (severity) and sensation of rigidity (extent) in the acupuncture treatment group, but not in the sham group. However, the biological mechanisms underlying the treatment of DPN by moxibustion have yet to be fully elucidated.

Persistent hyperglycemia is possibly the main cause of neuroinflammation and nerve damage leading to neuropathic pain. *Nrf2* is a redox-regulated transcription factor involved in the modulation of antioxidant defense systems. *Nrf2* stimulates the production of endogenous antioxidant defenses and detoxifying enzymes. *NF-κB* is a transcription factor involved in proinflammatory cytokine production, in addition to its immunological function. The regulation of *Nrf2* is coordinated with that of *NF-κB* to maintain redox homeostasis in healthy cells. However, this regulation is perturbed under pathological conditions; as such, an opportunity for therapeutic intervention becomes evident. Diabetic neuropathy is a condition, in which change in the expression pattern of *Nrf2* and *NF-κB* has been reported [22].

In our study, a rat model of DPN was established and histological changes in periodontal tissues were observed under an ultrascope. Nerve conduction indicators were detected with the electrophysiological method. The expression levels of *NF-κB* and *Nrf2* were observed through immunoblot. Our study aimed to investigate the role of *Nrf2* and *NF-κB* in diabetic neuropathy and to summarize the therapeutic outcomes of moxibustion targeted at *Nrf2*–*NF-κB* in diabetic neuropathy.

## Materials and Methods

### Reagents and Animals

Three-month-old male Wistar rats with a body weight of 200–220 g were purchased from Shanghai Slaccas Experimental Animal Co., Ltd. (Shanghai, China; Certificate no. SCXK 2015-0012). Moxibustion was purchased from the National Institute for the Control of Pharmaceutical and Biological Products (Beijing, China). All of the rats were provided free access to water and food and maintained in a 12 h:12 h light/dark cycle at 22 ± 2 °C and 65–69% relative humidity for 8 weeks. This study was approved by the ethics committee of Hubei University of Chinese Medicine (Wuhan, China). The animal research protocol was conducted in accordance with the European Community guidelines for the use of experimental animals. STZ was purchased from Hangzhou Baitong Biological Technology Co., Ltd. (Hangzhou, China). IL-1β, IL-6, and IL-8 ELISA commercially available kits (R&D Systems, Minneapolis, MN, USA) were used. Rabbit antibody against β-actin (ab189073), rabbit anti-*NF-κB* polyclonal antibody (ab7971), and rabbit anti-*Nrf2* polyclonal antibody (ab31163) were purchased from Abcam (Cambridge, MA, USA). Total RNA was extracted from freshly frozen neural tissues by using an Ultrapure RNA kit (CWbio Co., Ltd., China) and then reverse-transcribed with a HiFi-MMLV cDNA kit (CWbio Co., Ltd., China). Real-time PCR was performed in a Bioer line gene PCR instrument (BIOER, China) by using Invitrogen primers.

### Animal Groups and Model

In this experiment, 100 rats were used. After the rats were subjected to fasting overnight, diabetes was induced to 80 rats by intraperitoneally injecting STZ dissolved in 0.1 M sodium citrate buffer (pH 4.5) at a dose of 60 mg/kg [23]. The successful induction of diabetes was confirmed when fasting blood glucose exceeded 16.7 mmol/L 3 days after STZ was injected and remained at > 16.7 mmol/L throughout the study. In the normal control group (N), the 20 remaining rats were treated with the same volume of cold citrate buffer and considered as nondiabetic rats.

Ischemia-reperfusion was induced to the diabetic rats in the DPN model group, as previously described [24]. In brief, the STZ-diabetic rats were anesthetized by intraperitoneally administering 50 mg/kg soluble pentobarbital sodium [25] after 4 weeks of induction. Ischemia was induced by occluding the abdominal aorta, right common iliac artery, and femoral artery with artery clips, which were removed after 3 h. Sixty-three rats were included in the final study conducted for 4 weeks. Seventeen rats were excluded from the total 80 rats because of death during surgery due to infection (*n =* 5, the percentage is 6.25%) or because of an insufficient increase in fasting blood glucose (< 16.7 mmol/L; *n =* 12, the percentage is 15% ), which is almost similar to the result from the previous experiment [26]. During the last week after infection, every cage received hydrated gel (Clear H_2_O, Portland, ME), a solid form of fluid replacer that was maintained off the bedding in a disposable dish. Topical antibiotic ointment (Antibiotic Ointment, CVS Pharmacy brand) was applied to any rat that rat tail and toe joints developed erosion or even necrosis. Surviving rats were not expected to exhibit additional health concerns and therefore were checked daily by animal care staff until the final experiment. Any rat that experienced prolonged inactivity or moribundity (pale, tachypnea, cold and transparent ears, corneal opacity and dull eyes) was euthanized by CO_2_ narcosis, and any rat that spontaneously died due to infection were removed immediately from the cages. We retained 20% CO_2_ in the enclosed flow cage (30.5 cm in width × 30.5 cm in. height × 61 cm in length) for euthanasia of rats. The animal experiment protocol was approved by the Animal Ethics Committee of Hubei University of Chinese Medicine in China.

### Animal Treatment

Acupuncture points were referred to in the “Map of the Experimental Animal Acupuncture Points” developed by the Experimental Acupuncture Institute of China Association of Acupuncture and Moxibustion. Moxibustion was applied to the rats with DPN in the morning by Jia Li, who is a Doctor of Philosophy major in Acupuncture and Moxibustion. The rats were administered with mild anesthesia of 300 mg/kg chloral hydrate (including normal control and model groups, 4 ml/kg, less than the normal dose of 7 ml/kg); as a result, the rats could crawl after appropriate stimulation was administered. The rats completely recovered from the effects of anesthesia within 30 min. These rats were divided into three groups: DPN group (DPN, *n =* 23), DPN + moxibustion with 15 min/day group (M1, *n =* 19), and DPN + moxibustion with 30 min/day group (M2, *n =* 21). The moxibustion group were treated with moxibustion at “Zusanli” (ST 36), “Guanyuan” (BL 26), and “Yishu” (EX-B3) from the 4th week to the 8th week with a grain-sized moxa cone of approximately the size of a wheat grain (1 mg of pure moxa punk with a base of 2.5–3.0 mm and a height of 4–5 mm), once a day for 4 weeks at a rate of 15 and 30 min/day separately in M1 and M2 groups, respectively (the burning time of each cone was 10–12 s, with a temperature of 48–52 °C at acupoints, and the cone was removed before burning out in order to avoid the pain caused by heat stimulation in rats). Briefly, a grain-sized moxa cone was put on the acupoints using tweezers and ignited with a line-incense stick. A new moxa cone was applied when the prior one was burned up.

### Electrophysiology

Sciatic nerve conduction velocity (SNCV), nerve action potential (NAP) amplitude, and tail nerve conduction velocity (TNCV) were measured by using the BL-420F biological function experimental system (Chengdu Technology & Market Co., Ltd., China) with stimulating and recording electrodes. After the rats were anesthetized with a dose of 300 mg/kg chloral hydrate, the right sciatic nerves were exposed by blunt dissection. The nerve was stimulated proximally and the electric signal was recorded from the distal digital nerve. The TNCV was measured similarly by using single needle stimulating electrodes inserted into the proximal tail and the recording electrodes inserted distally. The time (*T*) and distance (*S*) between the stimulating electrodes and the recording electrodes were used to calculate CV expressed as follows: CV = *S*/*T*.

### Ultrastructural Observation

For histological analysis, the rats were anesthetized by intraperitoneally injecting with a dose of 300 mg/kg chloral hydrate and quickly decapitated. The sciatic nerve was removed at about 1 cm away from the sections used for the electrophysiology tests. The tissues were rapidly fixed with 2.5% pre-cooled glutaraldehyde in 0.1 M phosphate-buffered saline for 1–2 h, trimmed, and located. The suspension was mixed with 1% osmium tetrachloride for 1.5 h, dehydrated, embedded, sectioned, and examined using a Hitachi H-600 transmission electron microscope (Hitachi Company, Tokyo, Japan).

### Determination of the Serum Levels of Inflammatory Cytokines Through ELISA

Serum was isolated from the blood samples through centrifugation at 10000 rpm for 10 min and stored at − 20 °C prior to the assays. The serum levels of IL-1β, IL-6, and IL-8 cytokines were evaluated using specific ELISA kits in accordance with the manufacturer’s protocol.

### Immunoblot Analysis

Quick-frozen sciatic nerve samples were homogenized in dry ice and boiled in 50 μl of SDS sample buffer (1.5% dithiothreitol, 2% SDS, 80 mM Tris-HCl (pH 6.8), 10% glycerol, and 0.01% bromophenol blue) for 5 min and then separated through SDS-polyacrylamide gel electrophoresis (SDS-PAGE). After protein was transferred, the membrane was blocked with bovine serum albumin or milk for 1 h and probed by using a primary antibody, followed by horseradish peroxidase-conjugated secondary antibody. The levels of *NF-κB* and *Nrf2* protein were quantified through the scanning densitometry of immunoblots (Fuji Multigauge software).

### Real-time PCR

The sciatic nerve tissues were subjected to quantitative RT-PCR experiments. Total RNA was isolated from maxilla tissues by using Trizol reagent in accordance with the manufacturer’s protocol. The extracted RNA (1 μg) was reverse-transcribed into cDNA by using a PrimeScript RT reagent kit. RT-PCR was performed and from each PCR reaction. The primer sequences of *NF-κB*, *Nrf2*, and GAPDH were designed as follows: *NF-κB* p65, FW: 5′-GACGAGGCTCGGAGAGCCCA-3′ and RV: 5′-CTGGGGCGGCTGACCGAATG-3′; *Nrf2*, FW: 5′-TCCATTTCCGAGTCACTGAACCCA-3′ and RV: 5′-TGACTCTGACTCCGGCATTTCACT-3′; GAPDH, FW: 5′-CCTGGAGAAACCTGCCAAG-3′ and RV: 5′-CACAGGAGACAACCTGGTCC-3′. After PCR was completed, the products were analyzed through electrophoresis on 1.2% agarose gel and photographed under UV light in an EC3 Imaging System (UVP, Upland, CA).

### Data Processing

All statistical analysis was performed using Prism 6 software (GraphPad Software, San Diego, CA). Differences between pairs of groups were analyzed by the Student–Newman–Keuls test or Dunn’s method. Values of n refer to the number of experiments used to obtain each value. *p* < 0.05 was considered to be significant. PS: Power and Sample Size Calculation software (Vanderbilt University) was used to determine the sample size.

## Results

### Effects of Moxibustion on the Nerve Conduction Velocity of the Rats with DPN

**Fig. 1:**
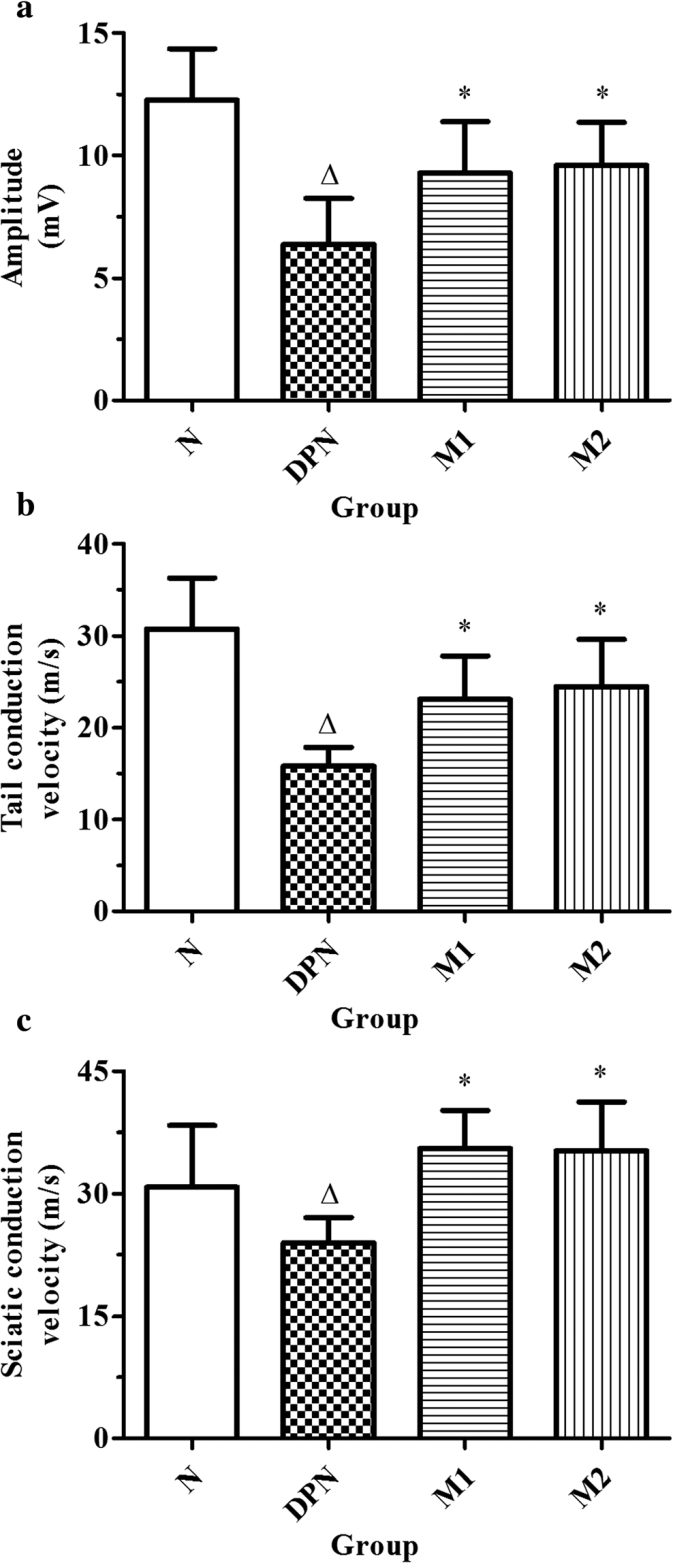
Action potential amplitude (**a**), sciatic nerve conduction velocity (**b**), and tail nerve conduction velocity (**c**) in N (normal control, *n =* 20), DPN (model, *n =* 23), M1 (moxibustion for 15 min/day, *n =* 19), and M2 (moxibustion for 30 min/day, *n =* 21) groups (∆*p* < 0.05 versus the normal control group, *p < 0.05 versus the model group)

To determine the potential therapeutic effect of moxibustion on DPN, we initially identified the effect of moxibustion on the nerve conduction of the rats with DPN. Compared with those of the normal control (N) group, the SNCV, NAP amplitude, and TNCV were significantly reduced in the DPN model (DPN) group [N versus DPN; SNCV (m/s): 44.23 ± 5.3 versus 25.18 ± 3.35; NAP amplitude (mV): 12.2 ± 2.42 versus 6.25 ± 1.08; TNCV: 31.46 ± 6.14 versus 16.27 ± 2.83; *n =* 20 in group N while *n* = 23 in Group DPN, *p* < 0.01]. The SNCV, NAP amplitude, and TNCV increased with moxibustion treatment of either 15 min/day (M1, *n* = 19) or 30 min/day (M2, *n* = 21), compared with those detected in the DPN group (*p* < 0.05 for both comparisons; Fig. 1). There was no significant difference between groups M1 and M2.

### Sciatic Nerve Ultrastructure

**Fig. 2:**
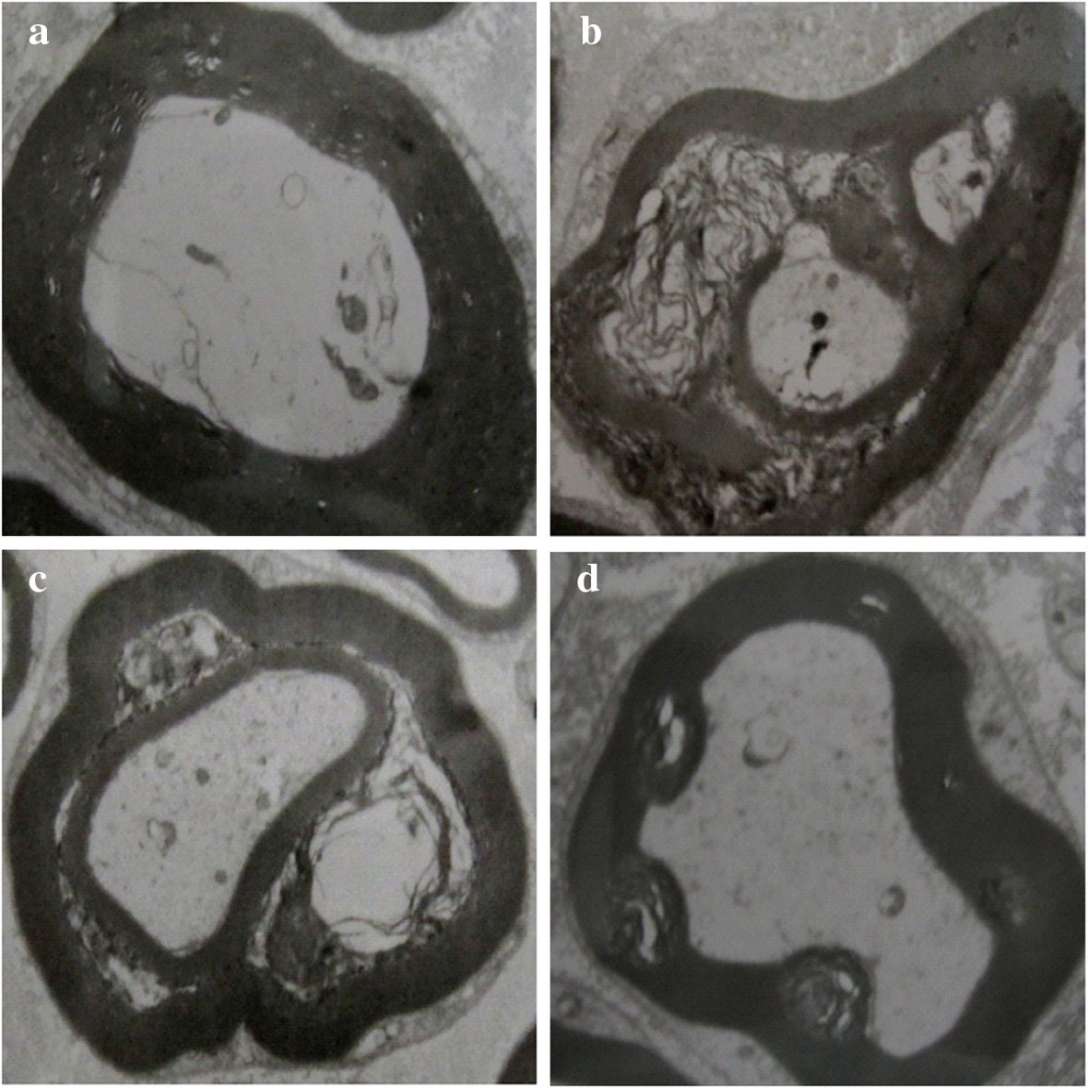
Sciatic nerve ultrastructure (uranium-lead staining). Normal control group (N) (**a**): sciatic nerve with intact myelin sheaths and healthy axons with prolific cell organelles. Model group (DPN) (**b**): the sciatic nerve with severe loss of myelinated axons and increased endoneurial collagen, disrupted and convoluted myelin sheaths, and decreased cell organelles. M1 (**c**): moxibustion for 15 min/day. M2 (d): moxibustion for 30 min/day

Considering that moxibustion treatment improves the NCV of the rats with DPN, we hypothesized that moxibustion may protect the ultrastructure of the nerve tissue against diabetic and ischemic insults. The ultrastructure of the sciatic nerves was visualized by using a transmission electron microscope. The figures show the representative ultrastructure features in the N group: × 6000, figure a; the DPN group: × 6000, figure b; the M1 group: × 6000, figure c; and the M2 group: × 6000, figure d. Figure 2 shows the typical electron microscopy results that confirmed the edematous and ischemic nerve myelin. Figure 2 also reveals myelin sheath swelling and disruption, axonal atrophy, and cellular organelle reduction in the DPN model group. After moxibustion treatment was administered for 15 or 30 min, the pathological changes in the treated rats were reduced compared with those in the normal control group. The pathological changes included the mitigation of edema and injury to myelin sheath and axons.

### Expression Levels of Serum IL-1β, IL-6, and IL-8

**Fig. 3:**
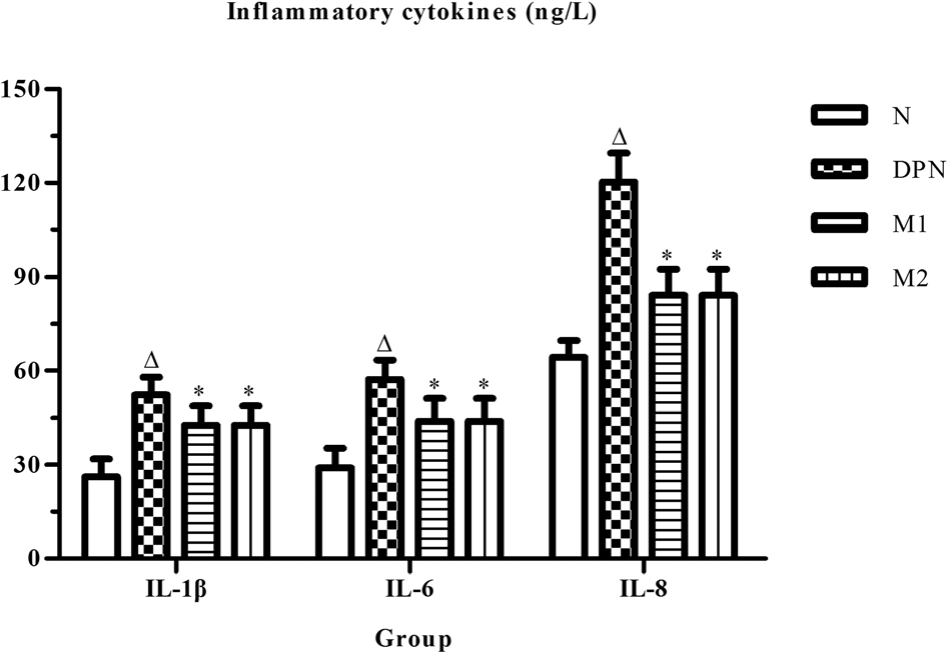
IL-1β, IL-6, and IL-8 contents in the plasma from N (normal control, *n =* 20), DPN (model, *n =* 23), M1 (moxibustion for 15 min/day, *n =* 21), and M2 (moxibustion for 30 min/day, *n =* 21) groups (∆p < 0.05 versus the normal control group, *p < 0.05 versus the model group).

ELISA analysis demonstrated that the expression levels of serum IL-1β, IL-6, and IL-8 in the rat model significantly increased compared with the corresponding values in the DPN group (*n =* 23, *p* < 0.01). The moxibustion treatment significantly decreased the expression levels of IL-1β, IL-6, and IL-8 in the M1 and M2 groups compared with those in the DPN groups (*n =* 19 in group M1 while *n* = 21 in group M2, *p* < 0.05). No changes were observed in the moxibustion group (Fig. 3).

### Immunoblot of the Sciatic Nerve

**Fig. 4:**
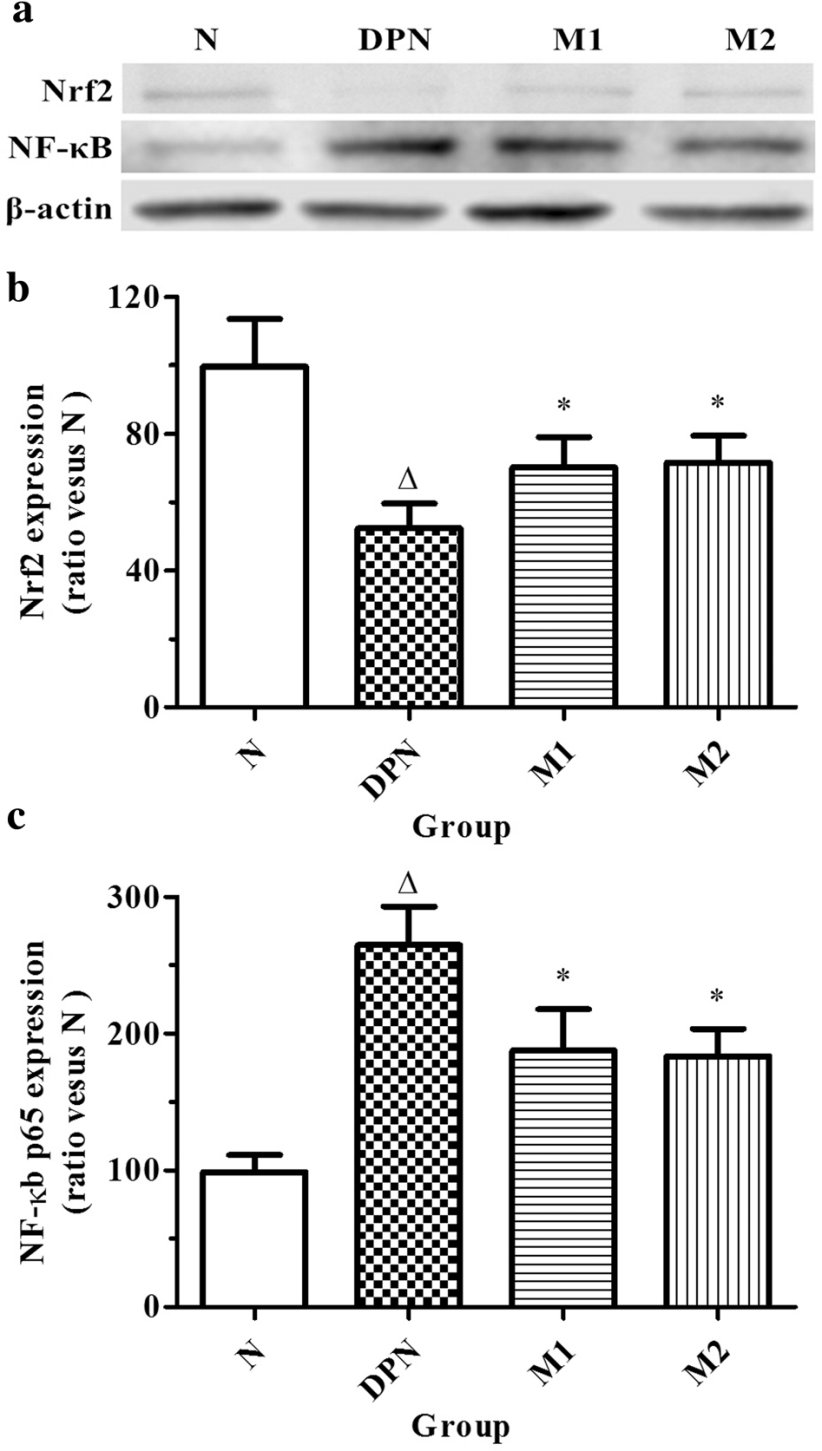
Expression level of *NF-κB* and *Nrf2* in maxilla from N (normal control, *n =* 20), DPN (model, *n =* 23), M1 (moxibustion for 15 min/day, *n =* 19), and M2 (moxibustion for 30 min/d, *n =* 21) groups (∆*p* < 0.05 versus the normal control group, *p < 0.05 versus the model group)

We determined the protein expression of *NF-κB* and *Nrf2* in the sciatic nerve to confirm the data obtained from previous experiments. Compared with group N (*n =* 20), the expression level of *NF-κB* in the sciatic samples from the DPN group was significantly increased, whereas the expression level of *Nrf2* significantly decreased (*n =* 23, *p* < 0.01). After the rats were treated with moxibustion at different durations, the expression levels of *NF-κB* in the sciatic nerve was significantly reduced, whereas the expression level of *Nrf2* significantly increased in the moxibustion groups compared with the DPN group (*n =* 19 in group M1 while *n* = 21 in group M2, *p* < 0.05). No significant difference was observed between M1 and M2 groups (Fig. 4).

### RT-PCR of the Sciatic Nerve

**Fig. 5:**
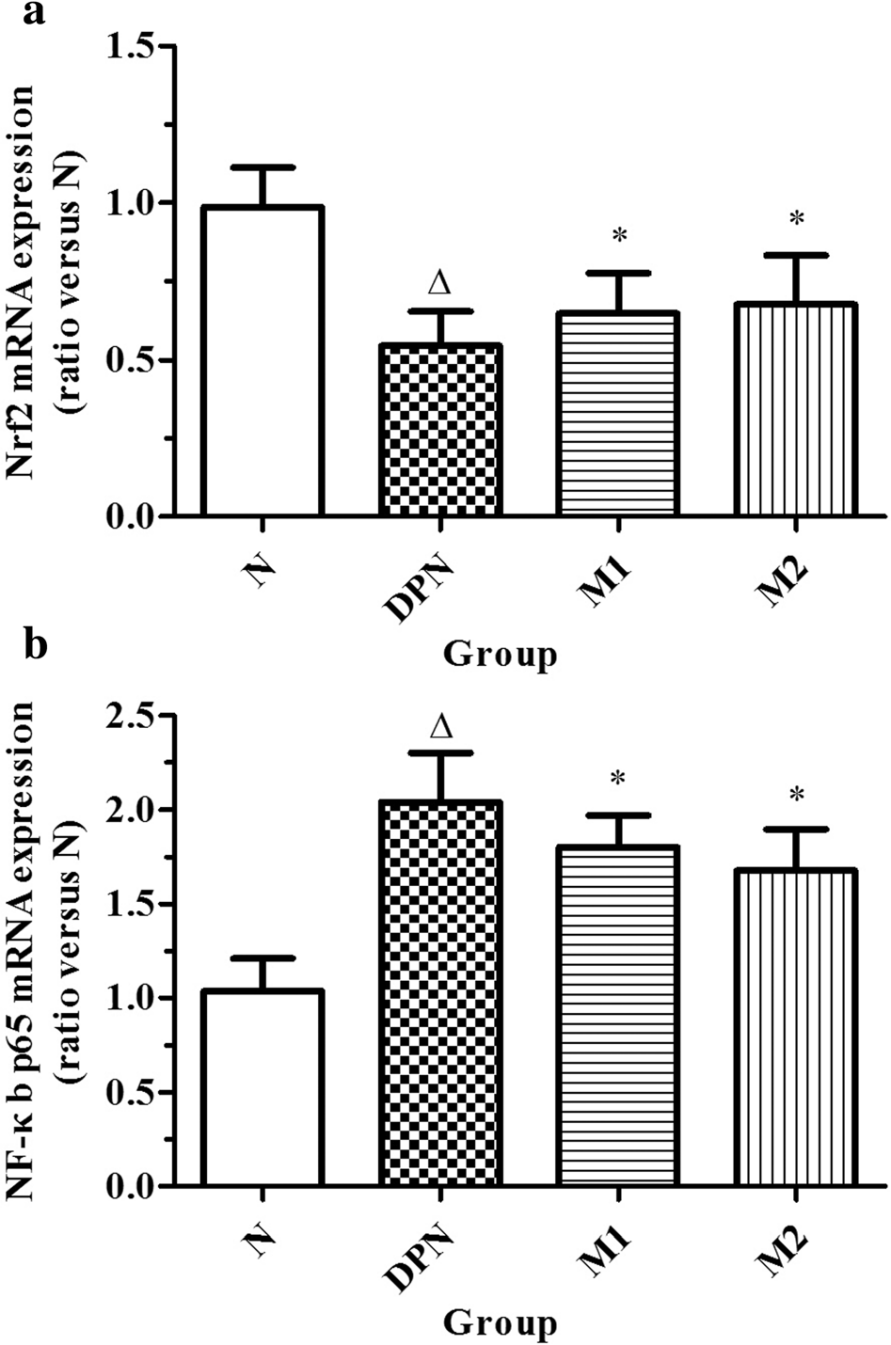
Real-time PCR of the sciatic nerve from N (normal control, *n =* 20), DPN (model, *n =* 23), M1 (moxibustion for 15 min/day, *n =* 19), and M2 (moxibustion for 30 min/day, *n =* 21) groups (∆*p* < 0.05 versus the normal control group, *p < 0.05 versus the model group).

To confirm the observations obtained from the immunoblot experiment, we further investigated the mRNA expression of *NF-κB* and *Nrf2*. Compared with the N group (*n =* 20), the mRNA expression level of *NF-κB* in the sciatic nerve in the DPN group was significantly increased (*n =* 23, *p* < 0.05). After the moxibustion treatment was administered, the mRNA expression levels of *NF-κB* in the sciatic nerve were significantly decreased in the moxibustion groups compared with the model group (*n =* 19 in Group M1 while n = 21 in group M2, *p* < 0.05). By contrast, the mRNA expression of *Nrf2* was decreased in the DPN group compared with the normal control group but relatively increased in the moxibustion-treated groups. However, no significant difference was observed between M1 and M2 groups (Fig. 5).

## Discussion

DPN accounts for most common long-term complications of diabetes mellitus. As such, neural cells play an important role in the pathogenesis of DPN. Neural deficits are associated with abnormal reactions of glial cells and neurons in response to hyperglycemia [27]. Moxibustion is administered as an effective and safe treatment for DPN [28, 29]; however, the therapeutic mechanism has yet to be fully elucidated. In this study, neural ultrastructural changes and conduction in the sciatic nerve of the rats were quantified after DPN was induced. Whether these changes are associated with *NF-κB* and *Nrf2* during moxibustion treatment was also investigated.

STZ is a broad-spectrum antibiotic, which is widely administered to experimental animals to induce diabetes; this antibiotic elicits toxic effects on the pancreatic β cells of pancreatic islets and thus causes the loss of insulin secretion of the islets [30]. STZ also triggers an immune response that leads to diabetes [31, 32]. The animals used to establish the model are usually inbred Wistar rats or Sprague-Dawley rats. Daniel *et al.* [33] reported that the success rate of the model establishment in male rats is significantly higher than that in female rats. Monea *et al.* [34] demonstrated that the sensitivity of rats to STZ occurs in an age-dependent manner. Our study used male Wistar rats with an average body weight of 212.5 g. After the rats were subjected to fasting overnight, diabetes was induced by intraperitoneally injecting 60 mg/kg STZ diluted in citrate buffer (pH 4.4). STZ-induced diabetes is characterized by hyperglycemia, severe body weight loss, polydipsia, polyphagia, and polyuria. Three days after STZ was injected, the blood glucose levels were assessed and the rats with blood glucose levels of > 16.7 mmol/l were considered diabetic. Compared with the normal rats, the STZ-treated rats yielded significantly increased blood glucose levels, reduced body weights, increased water and food consumption, and increased urine output (data not shown). These results confirmed that diabetes was effectively induced.

As a common complication of diabetes, DPN may lead to diabetic foot ulcers, even to foot or limb amputations. The first step in the treatment of DPN is prevention. DPN cannot be cured, and nerve damage cannot be repaired; therefore, individuals with diabetes must prevent the occurrence of DPN. No approved treatments have been administered to prevent or cure diabetic neuropathy, although symptomatic pain therapies of variable efficacy are available. In early 1987, WHO proposed 43 kinds of acupunctural indications, in which peripheral neuropathy was included. The traditional duration of a moxibustion treatment is 15 min and is targeted to fixed acupoints. Although some randomized and controlled trials have confirmed the efficiency of traditional moxibustion [35], strong evidence supporting the therapeutic effectiveness of this treatment is insufficient. Specific acupoints on the body are treated with moxibustion; thus, this treatment can produce the effect to regulate *qi* and blood, dredge the meridians, and balance the functions [36]. This treatment can also promote the elimination of contaminants and alleviate the pathological effects, such as inflammation, adhesion, exudation, and hematoma [37]. The duration of moxibustion treatment is often longer than that of normal treatment because duration is an important factor affecting the efficacy of moxibustion [38]. Therefore, this experiment administered 15- and 30-min moxibustion treatments. The results revealed that the application of moxibustion for 15 and 30 min/day significantly improved the neural conduction and alleviated the inflammation of the rats with DPN after 4 weeks of treatment. However, the difference between the two treatment durations was not significant. These results suggested that the 15-min moxibustion treatment sufficiently improves DPN.

Acupuncture and moxibustion preventive treatment for disease, which is the important component of the prevention treatment for disease in TCM, has widespread application since ancient times. Acupuncture can improve the ability to resist disease and can restore the ability to maintain the body’s steady state. This is also the recruitment of Chinese medicine “keep healthy, do not be evil.” Points ST 36 and BL 26 have a close relationship with congenital kidney and aquired spleen and stomach *qi* respectively, while EX-B 3 eliminate unhealthy trends. The point combination tends to address both the symptoms and root causes by paying equal attention to prevention.

*NF-κB* and *Nrf2* pathways mediate cellular homeostasis by controlling oxidative stress and inflammation. *NF-κB* suppresses the transcriptional activity of *Nrf2*. In summary, *NF-κB* and *Nrf2* individually affect many signaling cascades to maintain a redox homeostasis; these factors interact with each other to further modulate the level of key redox modulators of health conditions and diseases [39]. An imbalance between *NF-κB* and *Nrf2* is a key step in the disruption of diabetes and is a major contributor to the development and progression of DPN. Previous studies supported the defensive role of *Nrf2* in neurons exposed to oxidative stress and suggested that the *NF-κB* pathway is an important modulator of inflammatory damage in diabetic neuropathy [40]. Li *et al.* [41] demonstrated that *Nrf2*-deficient mice exhibit a greater induction of *NF-κB*-regulated pro-inflammatory genes, such as interleukins and TNF-α; thus, *Nrf2* deficiency enhances *NF-κB*-mediated pro-inflammatory reactions. High glucose–induced reactive oxygen intermediate production and inflammatory damage are considered as contributors of nerve dysfunction and subsequent damage in diabetic neuropathy. Melatonin modulates neuroinflammation by decreasing the *NF-κB* activation cascade and regulates oxidative stress by increasing the *Nrf2* expression; melatonin may elicit a neuroprotective effect against diabetic neuropathy [42]. Further studies support the defensive role of *Nrf2* in neurons exposed to oxidative stress and suggest that the *NF-κB* pathway is an important modulator of inflammatory damage in diabetic neuropathy [43]. Diabetes involves chronic hyperglycemia caused by a range of genetic and environmental factors, and this condition is widely considered as a risk factor of periodontal disease. Previous studies also demonstrated that advanced glycosylation ends triggered by long-term hyperglycemia can stimulate phagocytes to release inflammatory cytokines, including tumor necrosis factor, IL-1β, and IL-6 [44]. Cytokines, such as IL-1β, IL-6, and IL-17, can sensitize the peripheral receptors and thus cause neuropathic injury [45].

The main strengths of this study have been indicated. To our knowledge, this study is the first to investigate the effects of moxibustion treatment on DPN; thus, this study provided preliminary results as a basis of future studies. The novel treatment strategy may have an important role in an experimental setting because current pharmacological approaches are insufficient for DPN-related neuroinflammation. We also obtained that the 15-min moxibustion treatment could sufficiently improve DPN,which significantly contributes to the existing literature.

Our study showed that moxibustion can regulate and restore the balance between the expression levels of *NF-κB* and *Nrf2* and the pathological changes in the periodontal tissues of diabetic rats. Moxibustion alleviated the degradation of periodontal tissues and suppressed the synthesis and secretion of interleukins, such as IL-1β, IL-6, and IL-8, in DPN rats. Moxibustion inhibited *NF-κB* and rescued the *Nrf2* expression in the sciatic nerve. In conclusion, moxibustion may elicit therapeutic effects that simultaneously target the *Nrf2* and *NF-κB* pathways in DPN.

One of the limitations of this study is that although the rats with DPN were treated for 4 weeks to assess their nerve conduction, no continuous measurement was conducted to evaluate the long-term effect of moxibustion on the balance between *NF-κB* and *Nrf2* expressions. Future studies should combine experimental evaluation with microscopic and molecular studies on nerve conduction to elucidate the long-term efficacy of moxibustion. Further studies should also elucidate whether moxibustion directly inhibits or regulates the body’s metabolic function by eliminating inflammatory factors and by indirectly regulating *NF-κB* and *Nrf2* pathway. Besides, a transition to clinical research on humans needs further investigation.

## Conclusions

**Fig. 6:**
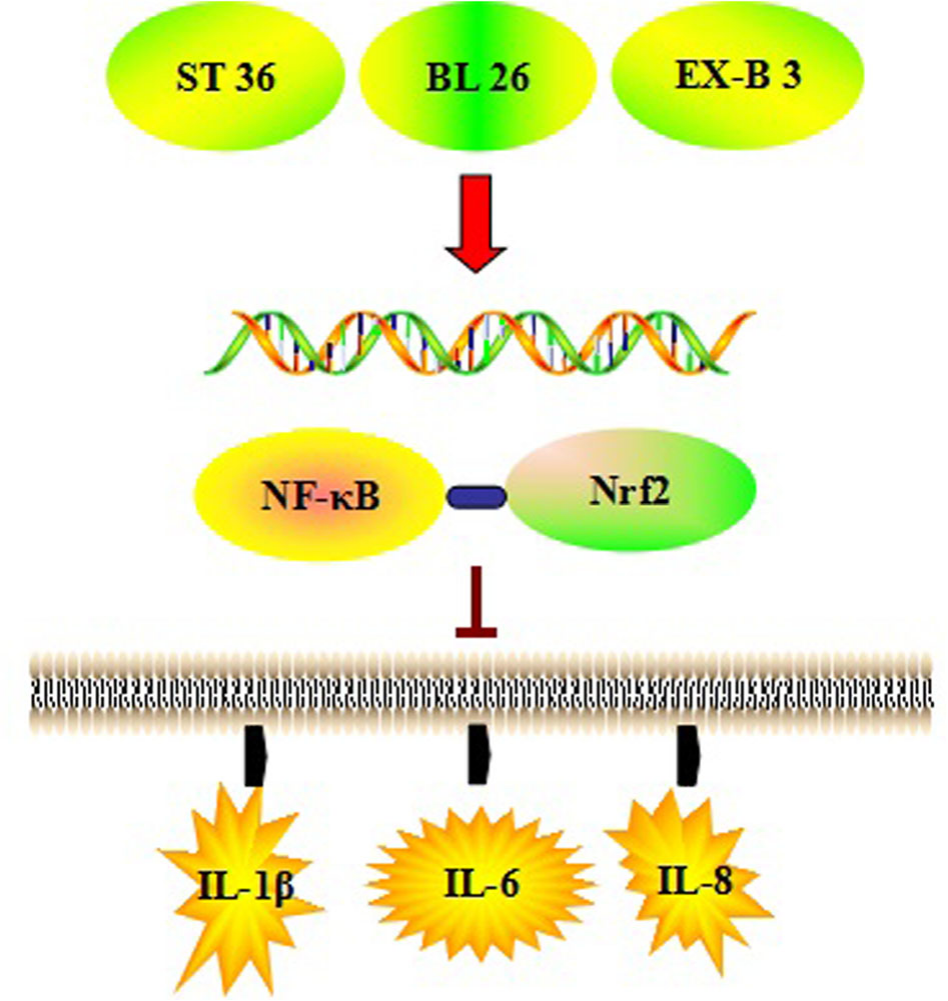
Moxibustion treatment on ST 36, BL 26, and EX-B 3 alleviates neuroinflammation via *NF-κB* inhibition and *Nrf2* activation

Our results show that moxibustion inhibited the protein and mRNA expression of *NF-κB* but induced the protein and mRNA expression of *Nrf2* in the sciatic nerve. Moxibustion restored the balance between *NF-κB* and *Nrf2* in rats with DPN. Thus, neural inflammation was relieved by regulating interleukin inflammatory factors [Fig. 6].
